# Cognitive impairment, mood, and fatigue in various multiple sclerosis subtypes: a one-year follow-up study

**DOI:** 10.1007/s00415-025-13115-y

**Published:** 2025-05-14

**Authors:** Daniela Taranu, Luisa T. Balz, Jill Holbrook, Visal Tumani, Herbert Schreiber, Hayrettin Tumani, Ingo Uttner

**Affiliations:** 1 Department of Neurology, Faculty of Medicine, Ulm University, D-89071 Ulm, Germany; 2 Department of Psychiatry, Faculty of Medicine, Ulm University, D-89071 Ulm, Germany; 3 Neurological Practice Center, Neuropoint Academy & NTD, D-89073 Ulm, Germany

**Keywords:** Multiple Sclerosis, Cognitive Impairment, Disease Progression, Psychopathology, Fatigue, Neuropsychological Assessment

## Abstract

**Background:**

Multiple sclerosis (MS) subtypes—relapsing–remitting (RRMS), secondary-progressive (SPMS), and primary-progressive (PPMS) – have been associated with distinct cognitive impairment profiles, with progressive subtypes, in contrast to RRMS, showing additional deficits in more widespread domains. Research has largely focused on RRMS, leaving SPMS and PPMS underexplored due to their lower prevalence and limited therapeutic targeting. Data on the interplay between cognitive impairment, mood, and fatigue over time are also scarce. This study examined cognition, fatigue, and psychopathology over a period of one year to identify subtype-specific impairments and progression trajectories.

**Methods:**

Sixty-six MS patients (22 each with RRMS, SPMS, and PPMS) and 22 healthy controls (HC) were assessed using neuropsychological tests for attention, memory, processing speed, working memory, fluency and visuospatial functions. Patient-reported outcomes for depression, anxiety, and fatigue were also collected. Analyses included correlations, within-group comparisons (paired t-tests), and between-group comparisons (ANOVAs/ANCOVAs).

**Results:**

Progressive MS subtypes exhibited more severe cognitive impairments, fatigue, and mood disturbances than RRMS. Over one year, treated RRMS patients improved in various cognitive domains, while PPMS patients showed gains only in visuospatial abilities. On the other hand, SPMS patients exhibited no significant changes, suggesting more pronounced cognitive deficits.

**Conclusions:**

Cognitive impairments differed significantly across MS subtypes. While RRMS patients improved over one year and PPMS patients showed selective gains in one domain, SPMS showed no significant changes, indicating reduced cognitive reserve. These between-group differences suggest different cognitive trajectories. The findings underscore the need for tailored, holistic interventions for different MS subtypes.

## Introduction

Multiple sclerosis (MS), an immune-mediated disorder characterized by inflammation and neurodegeneration [1], is the most common inflammatory disease of the central nervous system and the leading cause of permanent disability in young adults. Cognitive impairment is a frequent and often overlooked sequel of MS, significantly affecting patients’ daily functioning [1]. Cognitive deficits seem to vary across MS clinical phenotypes [2], which include relapsing–remitting MS (RRMS), secondary progressive MS (SPMS), and primary progressive MS (PPMS). RRMS is the most common phenotype, affecting 80–85% of patients [3]. Approximately 25–40% of RRMS cases convert to SPMS within two decades [3, 4] despite modern treatment interventions. In RRMS, cognitive impairment is reported in 21%−45% of patients [1, 4], mainly including reduced information processing speed and deficits in verbal fluency and visuospatial memory [4, 5]. In SPMS cognitive impairment affects up to 80% of patients [1, 4], with widespread deficits in information processing speed, verbal fluency, episodic memory, working memory, visuospatial abilities, and executive functions having been found [6, 7]. PPMS, affecting about 10–15% of MS patients, is typically marked by an insidious progression of disability without apparent relapses [8]. PPMS patients have originally been thought to be spared from cognitive impairment [9], but more refined assessments in more recent studies have provided convincing evidence that 56%−91% of PPMS patients experience cognitive deficits, particularly in attention, working memory, executive function, and verbal episodic memory [5, 10]. Despite the prevalence and impact of cognitive impairment in SPMS and PPMS, these patients are often underrepresented in scientific studies [11, 12], mainly due to lack of therapeutic targets against the pathohysiological drivers of progression, but also due to physical challenges posed by the progressive nature of these subtypes, which can make participation in longitudinal studies difficult [12]. Therefore, most studies focus on RRMS patients [13, 14], who experience milder symptoms. This imbalance leaves gaps in our understanding of cognitive changes, especially in SPMS and PPMS.

Recent studies by van Dam et al.[15], De Meo et al.[16], and Podda et al.[17] have also highlighted the variability of cognitive impairment in MS, identifying distinct cognitive profiles, particularly in progressive forms of the disease. These findings fuel and expand ongoing discussions about cognitive deficits in MS and raise the important question of how cognitive phenotypes differ depending on the underlying MS disease course.

Our study aims to characterize specific cognitive trajectories across MS clinical subtypes at baseline and after a one-year follow-up, including comparisons with healthy controls (HC). We additionally explore the interplay between fatigue, depression, and cognitive function, aiming to clarify how these factors contribute to cognitive symptoms across MS phenotypes. This approach offers a more detailed understanding of cognitive changes in MS, including characterization of non-motor clinical and behavioral symptoms affecting patients’ quality of life.

## Materials and methods

### Design and participants

Sixty-six MS patients (n = 22 each with RRMS, SPMS, and PPMS) were recruited from Ulm University’s Neurology Department, along with 22 age- and sex-matched HCs, primarily caregivers. All participants provided informed consent. The study, approved by Ulm University’s ethics committee (No.157/16), comprised a one-year prospective longitudinal design with data collected from 2017 to 2019. Inclusion criteria were confirmed MS diagnosis (revised McDonald criteria [18]), age 18–85, and ability to communicate during assessments. Exclusion criteria included motor, speech, or language impairments affecting test validity, complicating illnesses, psychiatric disorders, recent corticosteroid use (patients had to be stable for at least 30 days before data collection, with no corticosteroid treatment or relapse during this period), or unstable clinical status. No changes in symptomatic therapy (e.g., antidepressants or antispasmodics) were made in patients with psychological symptoms or fatigue, and no modifications were made to Disease-Modifying Therapies (DMTs) during the follow-up period.

### Procedure

All MS patients underwent neurological and neuropsychological assessments targeting memory, attention, executive, and visuospatial functions at baseline (T0) and after one year (T1). Depression, anxiety, and fatigue were evaluated using standardized patient-reported measures. Demographics and clinical data were collected via semi-structured interviews. Physical impairment was assessed with the Expanded Disability Status Scale (EDSS), ranging from 0 (normal function) to 10 (death) [19]. Parallel versions of the Symbol Digit Modalities Test (SDMT) [20], Brief Visuospatial Memory Test (BVMT-R) [21], and Verbal Learning and Memory Test (VLMT) [22] were used at follow-up to reduce practice effects.

### Cognitive assessments

The neuropsychological test battery included seven validated assessments: oral SDMT (information processing speed, attentional shift, visual scanning) [20], VLMT (verbal episodic memory), BVMT-R (visual episodic memory) [22], Paced Auditory Serial Addition Test (PASAT; auditory processing speed, working memory) [23], Controlled Oral Word Association Test (COWAT; verbal fluency) [24], and Block Design Test (BDT; visuospatial functions) [25].

### Depression, anxiety and fatigue

Depression and anxiety were assessed using the Hospital Anxiety and Depression Scale (HADS), a tool for individuals with physical illnesses with 2 subscales of 7 items (total possible score of 21 points) and cut-off scores for mild (≥ 8), moderate (≥ 11), or severe (≥ 15) [26] depression and anxiety, respectively. Fatigue was measured using the Fatigue Scale for Motor and Cognitive Functions (FSMC), a 20-item self-assessment questionnaire evaluating global, motor and cognitive fatigue, with subscale scores ranging from 10 to 50. Cut-off scores for global fatigue were ≥ 43 (mild), ≥ 53 (moderate), ≥ 63 (severe); for motor fatigue ≥ 22 (mild), ≥ 27 (moderate), ≥ 32 (severe); and for cognitive fatigue ≥ 22 (mild), moderate (≥ 28), severe (≥ 34) [27].

### Statistical analysis

Statistical analyses were conducted using SPSS version 29 and R. A priori power calculations ensured 80% power to detect small to large effect sizes (*r* = 0.1–0.3; Cohen’s d = 0.70) with α = 0.05, resulting in a required total sample size of 88 participants, with 22 per group. Chi-square tests assessed gender matching between MS patients and HC. ANCOVA (with age and education as covariates) and ANOVA compared groups at baseline and follow-up, with post-hoc tests (Tukey or Games-Howell for variance violations). Paired t-tests analyzed within-group changes. Correlations examined links between psychopathology, fatigue, and cognition. Data were z-standardized using the data of the 22 HC at baseline. Composite scores for global cognition, psychopathology, and fatigue were calculated by averaging z-standardized subscores, with psychopathology and fatigue scores inverted. Global cognition refers to a composite score that includes all z-standardized cognitive tests used in the study, summarized into this single composite measure.

## Results

### Demographics and clinical data

In sum, 88 participants were included, consisting of 22 patients with RRMS, 22 patients with SPMS, 22 patients with PPMS, and 22 HC. A significant age difference was observed among the four groups, with patients diagnosed with PPMS being the oldest. The groups were comparable in terms of gender and education. As expected, both time since disease onset and since diagnosis varied significantly across the MS subtypes, with SPMS patients exhibiting the longest disease duration. SPMS patients also demonstrated the highest level of physical impairment, as indicated by an average EDSS score of 5.96, reflecting moderate to severe disability. Demographic and clinical data are summarized in Table 1.

**Table 1:** Demographics and clinical data of MS patients and HC

Characteristics	RRMS (*N* = 22)		SPMS (*N* = 22)		PPMS (*N* = 22)		HC (*N* = 22)		Statistics
	Mean/N	SD/%	Mean/N	SD/%	Mean/N	SD/%	Mean/N	SD/%	
Age (years)	40.23	10.00	51.41	8.78	53.27	6.64	49.82	14.81	***F*(3, 84) = 6.75,** ***p* < 0.001**
Female	15	68.2%	12	54.5%	14	63.6%	11	50%	χ^2^(3) = 1.88, *p* =.598
Male	7	31.8%	10	45.5%	8	36.4%	11	50%	
Education (years)	10.32	2.53	10.09	2.71	11.18	1.89	11.82	2.22	*F*(3, 84) = 2.51, *p* =.064
EDSS	2.61	1.47	5.96	1.07	4.61	1.72			
EDSS 0–3.0	18	81.8%	0	0%	4	18.2%			
EDSS 3.5–6.0	3	13.6%	10	45.5%	11	50.0%			
EDSS ≥ 6.5	1	4.5%	12	54.5%	7	31.8%			
Time since onset (years)	11.82	8.75	18.23	8.30	10.82	7.25			***F*(2, 63) = 5.39,** ***p* =.007**
Time since diagnosis (years)	8.18	6.22	14.64	8.09	6.14	4.49			***F*(2, 63) = 10.4, *p* < 0.001**
Current therapy									
No therapy	5	22.7%	9	40.9%	12	54.6%			
First line	13	59.1%	5	22.7%					
Second line	4	18.2%	3	13.6%					
Third line			2	9.1%	7	31.8%			
Biotin			3	13.6%	3	13.6%			

First line therapies: interferons, glatiramer acetate, teriflunomide, dimethyl fumarate. Second line therapies: fingolimod, siponimod, cladribine. Third line therapies: natalizumab, ocrelizumab

*RRMS* Relapsing Remitting Multiple Sclerosis. *SPMS* Secondary Progressive Multiple Sclerosis. *PPMS* Primary Progressive Mulitple Sclerosis. *HC* Healthy Controls. *EDSS* Expanded Disability Status Scale

The use of bold formatting indicates statistical significance

### Between-group comparison and correlations at baseline

**Fig. 1:**
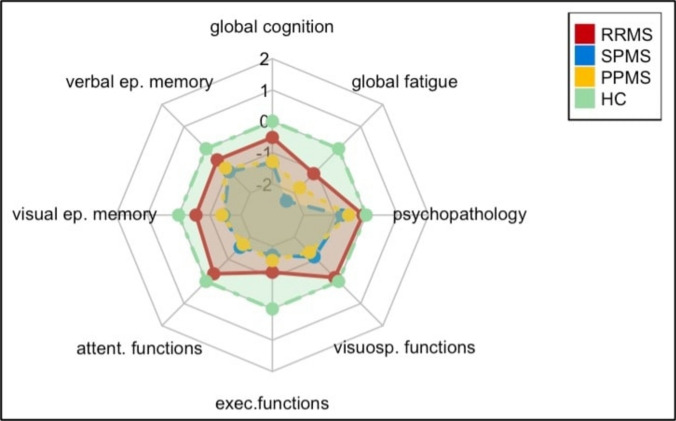
Between-group comparison at baseline

HC outperformed all MS subtypes across most cognitive domains, even after adjusting for age and education (Table 2). Composite z-scores revealed significant group differences when comparing RRMS with a combined group of progressive MS forms (SPMS and PPMS, N = 44). Patients with progressive MS performed significantly worse than those with RRMS in global cognition, visual episodic memory, attentional functions, visuospatial functions, global fatigue, and psychopathology (Δ [− 1.26, − 0.57], *p* [0.001, 0.049]). When analyzing at the subtype level and considering SPMS and PPMS separately (N = 22 respectively) SPMS (Δ = − 1.32, *p* < 0.001) and PPMS (PPMS: Δ = − 1.29, *p* < 0.001) showed significantly worse global cognition than HC, but not RRMS. Additionally, PPMS (Δ = − 0.77, *p* = 0.040) scored lower than RRMS. With respect to cognitive profile, RRMS (Δ = − 1.04, *p* = 0.015) and both progressive forms (SPMS: Δ = − 1.45, *p* = 0.002; PPMS: Δ = − 1.39, *p* = 0.003) performed poorly in verbal and visual episodic memory in comparison with HC. Attention deficits were prominent in SPMS (Δ = − 1.54, *p* = 0.009) and PPMS (Δ = − 1.67, *p* = 0.002) compared with HC, with PPMS (Δ = − 1.33, *p* = 0.027) scoring lower than RRMS. SPMS patients (Δ = 0.77, *p* = 0.030) reported higher psychopathology. Fatigue was pronounced across all MS subtypes (SPMS: Δ = 2.37, *p* < 0.001; PPMS: Δ = 1.76, *p* < 0.001; RRMS: Δ = 1.13, *p* = 0.010), and SPMS showed higher fatigue than RRMS (Δ = − 1.24, *p* = 0.004). Executive function deficits were significant in all MS groups, but visuospatial impairments were more severe in progressive forms (SPMS: Δ = − 1.11, *p* = 0.002; PPMS: Δ = − 1.31, *p* < 0.001) (Fig. 1).

**Table 2:** Cognitive assessment, psychopathology and fatigue of MS patients and HC (at baseline)

Cognitive domains/tests	RRMS (*N* = 22)		SPMS (*N* = 22)		PPMS (*N* = 22)		HC (*N* = 22)		Statistics	Statistics
	Mean/N	SD/%	Mean/N	SD/%	Mean/N	SD/%	Mean/N	SD/%	Unadjusted	adjusted for age, education
Verbal episodic memory										
VLMT total	53.41	9.46	49.77	12.47	50.73	9.87	59.32	8.36	**F(3, 84) = 3.94, *p* = 0.011, partial η**^**2**^** = 0.123**	F(3, 82) = 2.48, *p* = 0.067, partial η^2^ = 0.083
VLMT delayed recall	11.00	2.81	9.23	3.61	10.64	3.27	12.82	1.92	**F(3, 84) = 5.44, *p* = 0.002, partial η**^**2**^** = 0.163**	**F(3, 82) = 3.73, *p* = 0.014, partial η**^**2**^** = 0.120**
VLMT recognition	14.18	1.22	13.86	1.75	13.41	1.74	14.00	1.27	F(3, 84) = 1.04, *p* =.379, partial η^2^ = 0.036	F(3, 82) = 0.51, *p* =.677, partial η^2^ = 0.018
Visual episodic memory										
BVMT-R total	24.18	6.23	20.09	8.23	18.95	7.27	26.45	5.71	**F(3, 84) = 5.62, *p* = 0.001, partial η**^**2**^** = 0.167**	**F(3, 82) = 3.68, *p* = 0.015, partial η**^**2**^** = 0.119**
BVMT-R delayed recall	9.32	2.21	7.55	2.99	8.09	2.47	10.50	1.66	**F(3, 84) = 6.76, *p* < 0.001, partial η**^**2**^** = 0.194**	**F(3, 82) = 4.70, *p* = 0.004, partial η**^**2**^** = 0.147**
Attentional functions										
PASAT-3 total	46.18	8.84	40.33	15.17	37.27	15.45	47.95	6.29	**F(3, 83) = 3.74, *p* = 0.014, partial η**^**2**^** = 0.119**	**F(3, 81) = 2.84, *p* = 0.043, partial η**^**2**^** = 0.095**
SDMT	53.09	9.86	41.91	15.08	42.95	13.00	56.45	8.21	**F(3, 84) = 8.29, *p* < 0.001, partial η**^**2**^** = 0.228**	**F(3, 82) = 5.54, *p* = 0.002, partial η**^**2**^** = 0.168**
Executive functions										
COWAT	30.91	9.17	26.95	9.10	28.18	9.90	39.45	7.28	**F(3, 84) = 8.79, *p* < 0.001, partial η**^**2**^** = 0.239**	**F(3, 82) = 7.78, *p* < 0.001, partial η**^**2**^** = 0.222**
Visuospatial functions										
BDT	50.23	9.35	40.64	10.91	38.55	10.67	52.00	10.24	**F(3, 84) = 9.42, *p* < 0.001, partial η**^**2**^** = 0.252**	**F(3, 82) = 6.17, *p* < 0.001, partial η**^**2**^** = 0.184**
Global Fatigue (FSMC)	54.95	19.52	73.95	13.49	64.86	21.45	37.77	14.79	**F(3, 84) = 17.01, *p* < 0.001,** **partial η**^**2**^** = 0.378**	
Mild	2	9.1%	2	9.1%	3	13.6%	4	18.2%		
Moderate	2	9.1%	3	13.6%	3	13.6%	3	13.6%		
Severe	10	45.5%	17	77.3%	13	59.1%	1	4.5%		
Cognitive Fatigue (FSMC)	27.09	10.11	34.45	9.23	29.18	11.96	19.23	7.18	**F(3, 84) = 9.21, *p* < 0.001,** **partial η**^**2**^** = 0.247**	
Mild	3	13.6%	3	13.6%	3	13.6%	4	18.2%		
Moderate	4	18.2%	6	27.3%	5	22.7%	3	13.6%		
Severe	7	31.8%	11	50%	8	36.4%	1	4.5%		
Motor Fatigue (FSMC)	27.86	9.81	39.50	5.36	35.68	10.92	18.55	8.02	**F(3, 84) = 24.49, *p* < 0.001,** **partial η**^**2**^** = 0.467**	
Mild	2	9.1%	0	0%	1	4.5%	4	18.2%		
Moderate	1	4.5%	2	9.1%	4	18.2%	2	9.1%		
Severe	11	50%	20	90.9%	15	68.2%	1	4.5%		
Depression (HADS-D)	3.36	2.48	6.91	3.99	5.55	3.13	3.82	3.26	**F(3, 84) = 5.52, *p* = 0.002,** **partial η**^**2**^** = 0.165**	
Mild	1	4.5%	6	27.3%	8	36.4%	3	13.6%		
Moderate	0	0%	5	22.7%	0	0%	0	0%		
Severe	0	0%	0	0%	0	0%	0	0%		
Anxiety (HADS-A)	5.82	3.30	6.68	3.86	6.64	3.43	4.73	3.25	F(3, 84) = 1.53, *p* = 0.212, partial η^2^ = 0.052	
Mild	4	18.2%	7	31.8%	2	9.1%	2	9.1%		
Moderate	2	9.1%	3	13.6%	4	18.2%	1	4.5%		
Severe	0	0%	0	0%	0	0%	0	0%		

*RRMS* Relapsing Remitting Multiple Sclerosis. *SPMS* Secondary Progressive Multiple Sclerosis. *PPMS* Primary Progressive Mulitple Sclerosis. *HC* Healthy Controls. *VLMT* Verbal Learning Memory Test. *BVMT-R* Brief Visuospatial Memory Test – Revised. *COWAT* Controlled Oral Word Association Test. *PASAT-3* Paced Auditory Serial Addition Test – 3 s version. *SDMT* Symbol Digit Modalities Test. *BDT* Block Design Test. *FSMC* Fatigue Scale for Motor and Cognitive Functions. *HADS* Hospital Anxiety and Depression Scale

The use of bold formatting indicates statistical significance

### Between-group comparison and correlations at follow-up

**Fig. 2:**
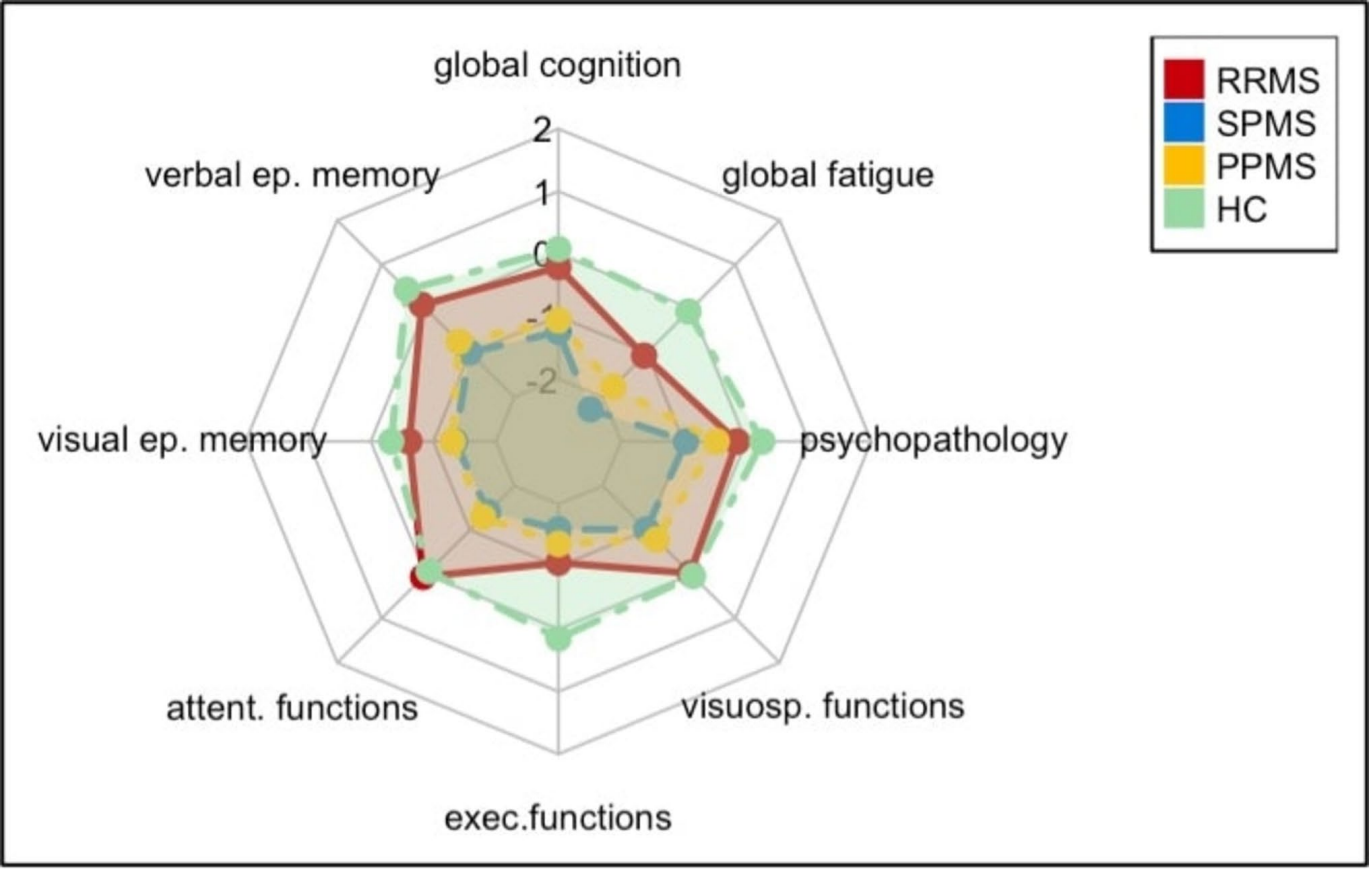
Between-group comparison at follow-up

Overall, 87 out of the initial 88 participants remained in the study from T0 to T1 (drop-out rate 1.14%). At follow-up, HC generally outperformed all MS subtypes, the most substantial differences being observed in verbal and visual episodic memory, attention, executive, and visuospatial functions (Table 3; Fig. 2). Even after adjustments for age and education, many of these differences persisted, particularly between HC and progressive forms of MS. Additionally, levels of cognitive and motor fatigue and depressive symptoms were significantly higher in progressive MS subtypes compared with HC (Table 3).

**Table 3:** Cognitive test results of MS patients and HC – follow-up (after 12 months)

Cognitive domains/tests	RRMS (*N* = 22)		SPMS (*N* = 22)		PPMS (*N* = 21)		HC (*N* = 22)		Statistics	
	Mean/N	SD/%	Mean/N	SD/%(sample size)	Mean/N	SD/%	Mean/N	SD/%	Unadjusted	Adjusted for age, education
Verbal episodic memory										
VLMT total	58.77	6.23	50.55	12.37	53.76	10.28	63.09	7.87	**F(3, 83) = 7.46, *p* <.001, partial η**^**2**^** =.212**	**F(3, 81) = 5.17, *p* =.003, partial η**^**2**^** =.161**
VLMT delayed recall	12.50	1.99	9.82	3.58	10.67	2.87	13.55	1.77	**F(3, 83) = 8.97, *p* <.001, partial η**^**2**^** =.245**	**F(3, 81) = 6.23, *p* <.001, partial η**^**2**^** =.187**
VLMT recognition	14.64	0.79	13.55	1.79	13.43	1.91	14.59	0.73	**F(3, 83) = 4.65, *p* =.005, partial η**^**2**^** =.144**	**F(3, 81) = 3.60, *p* =.017, partial η**^**2**^** =.118**
Visual episodic memory										
BVMT-R total	24.27	3.97	20.86	6.46 *(21)*	20.52	4.71	25.86	6.52	**F(3, 82) = 4.82, *p* =.004, partial η**^**2**^** =.150**	**F(3, 80) = 2.96, *p* =.037, partial η**^**2**^** =.100**
BVMT-R delayed recall	9.09	1.38	7.67	2.35 *(21)*	7.90	1.90	9.59	1.74	**F(3, 82) = 5.29, *p* =.002, partial η**^**2**^** =.162**	**F(3, 80) = 3.15, *p* =.030, partial η**^**2**^** =.106**
attentional functions										
PASAT-3 total	48.45	8.41	40.33	14.10 *(21)*	41.43	12.45	47.82	7.38	**F(3, 82) = 3.23, *p* =.026, partial η**^**2**^** =.106**	F(3, 80) = 1.90, *p* =.137, partial η^2^ =.066
SDMT	56.77	10.73	43.29	15.89 *(21)*	43.76	10.75	55.50	9.29	**F(3, 82) = 8.11, *p* <.001, partial η**^**2**^** =.229**	**F(3, 80) = 4.82, *p* =.004, partial η**^**2**^** =.153**
Executive functions										
COWAT	31.73	9.49	27.82	8.51	29.52	9.21	40.55	7.22	**F(3, 83) = 9.39, *p* <.001, partial η**^**2**^** =.253**	**F(3, 81) = 7.69, *p* <.001, partial η**^**2**^** =.222**
Visuospatial functions										
BDT	51.77	9.40	41.62	11.54 *(21)*	44.00	11.15	52.41	9.42	**F(3, 82) = 5.91, *p* =.001, partial η**^**2**^** =.178**	**F(3, 80) = 3.00, *p* =.035, partial η**^**2**^** =.101**
Global Fatigue (FSMC)	54.00	17.85	72.59	17.71	64.90	20.67	38.73	14.61	**F(3, 83) = 14.92, *p* <.001,** **partial η**^**2**^** =.350**	
Mild	5	22.7%	2	9.1%	3	13.6%	3	13.6%		
Moderate	2	9.1%	3	13.6%	4	18.2%	0	0%		
Severe	9	40.9%	16	72.7%	11	50.0%	3	13.6%		
Cognitive Fatigue (FSMC)	26.27	9.10	33.64	10.63	29.00	12.54	19.59	7.16	**F(3, 83) = 7.54, *p* <.001,** **partial η**^**2**^** =.214**	
Mild	5	22.7%	3	13.6%	4	18.2%	4	18.2%		
Moderate	3	13.6%	3	13.6%	2	9.1%	2	9.1%		
Severe	7	31.8%	13	59.1%	9	40.9%	1	4.5%		
Motor Fatigue (FSMC)	27.73	9.18	38.95	7.93	35.90	9.72	19.14	7.88	**F(3, 83) = 22.89, *p* <.001,** **partial η**^**2**^** =.453**	
Mild	2	9.1%	0	0%	1	4.5%	2	9.1%		
Moderate	4	18.2%	3	13.6%	4	18.2%	1	4.5%		
Severe	10	45.5%	18	81.8%	14	63.6%	3	13.6%		
Depression (HADS-D)	3.59	2.67	8.09	3.78	5.71	3.32	3.05	2.40	**F(3, 83) = 12.17, *p* <.001,** **partial η**^**2**^** =.306**	
Mild	1	4.5%	10	45.5%	6	27.3%	0	0%		
Moderate	1	4.5%	3	13.6%	2	9.1%	0	0%		
Severe	0	0%	1	4.5%	0	0%	0	0%		
Anxiety (HADS-A)	5.91	3.85	6.73	4.23	5.90	4.16	3.82	3.29	F(3, 83) = 2.23, *p* =.090, partial η^2^ =.075	
Mild	5	22.7%	1	4.5%	1	4.5%	0	0%		
Moderate	3	13.6%	7	31.8%	5	22.7%	0	0%		
Severe	0	0%	0	0%	0	0%	1	4.5%		

*RRMS* Relapsing Remitting Multiple Sclerosis. *SPMS* Secondary Progressive Multiple Sclerosis. *PPMS* Primary Progressive Mulitple Sclerosis. *HC* Healthy Controls. *VLMT* Verbal Learning Memory Test. *BVMT-R* Brief Visuospatial Memory Test – Revised. *COWAT* Controlled Oral Word Association Test. *PASAT-3* Paced Auditory Serial Addition Test – 3 s version. *SDMT* Symbol Digit Modalities Test. *BDT* Block Design Test. *FSMC* Fatigue Scale for Motor and Cognitive Functions. *HADS* Hospital Anxiety and Depression Scale

The use of bold formatting indicates statistical significance

Follow-up comparisons of z-standardized composite scores, initially conducted at the group level by combining SPMS and PPMS into a progressive MS group (N = 44) and comparing it to RRMS, revealed that patients with progressive MS exhibited significantly poorer performance across multiple domains. These included global cognition, visual and verbal episodic memory, attentional functions, visuospatial functions, and global fatigue (Δ [− 1.41, − 0.71], *p* [0.001, 0.022]). When analyzing at the subtype level, separating SPMS and PPMS and comparing each to RRMS, both SPMS (Δ = − 1.03, *p* = 0.007) and PPMS (Δ = − 0.85, *p* = 0.010) patients showed significantly lower global cognition scores than those with RRMS. Additionally, SPMS patients performed significantly worse in verbal episodic memory compared to RRMS (Δ = − 1.08, *p* = 0.009). With respect to attention, both SPMS (Δ = − 1.47, *p* = 0.022) and PPMS (Δ = − 1.35, *p* = 0.009) patients had significantly poorer results than RRMS patients. Psychopathology was also higher in the SPMS group (Δ = 0.82, *p* = 0.030) compared with RRMS. Visuospatial functions were impaired in patients with progressive MS, with SPMS patients (Δ = − 0.99, *p* = 0.010) showing lower scores than those with RRMS. Finally, fatigue levels were higher in SPMS patients (Δ = 1.21, *p* = 0.005) compared with RRMS (Fig. 2).

### Correlation analyses in MS subtypes at baseline and follow-up

At baseline, correlation analyses on data from RRMS patients revealed significant positive associations between psychopathology and fatigue (*r* = 0.57, *p* = 0.005) and between fatigue and global cognition (*r* = 0.45,* p* = 0.035). In PPMS patients, a significant correlation was likewise observed between psychopathology and fatigue (*r* = 0.66,* p* < 0.001), but not between fatigue and global cognition. For SPMS patients, no significant correlations were found (*p* > 0.05) between these domains.

At follow-up, correlation analyses indicated a significant association between fatigue and psychopathology for RRMS (*r* = 0.46, *p* = 0.030), SPMS (*r* = 0.51, *p* = 0.015), and PPMS (*r* = 0.86, *p* < 0.001). However, no significant correlations were found between global cognition and fatigue or psychopathology for any of the MS subtypes (all *p* > 0.05).

### Within-group comparison from baseline to follow-up

For within-group comparisons at one year compared with baseline, significant improvements were observed in the RRMS group in several cognitive domains. Specifically, RRMS patients showed significant gains in verbal episodic memory subtests, including total learning score (VLMT total: *t*(21) = − 3.75, *p* = 0.001, d = − 0.799) and delayed recall (VLMT recall: *t*(21) = − 3.05, *p* = 0.006, d = −0.651). Additionally, a significant improvement was noted in attentional function (PASAT: *t*(21) = − 3.52, *p* = 0.002, d = − 0.751). Composite scores showed RRMS patients achieving significant gains in global cognition (*t*(21) = − 5.09, *p* < 0.001, d = − 1.09), verbal episodic memory (*t*(21) = − 4.00, *p* < 0.001, d = −0.852), and attentional functions (*t*(21) = − 3.38, *p* = 0.003, d = − 0.72).

**Fig. 3:**
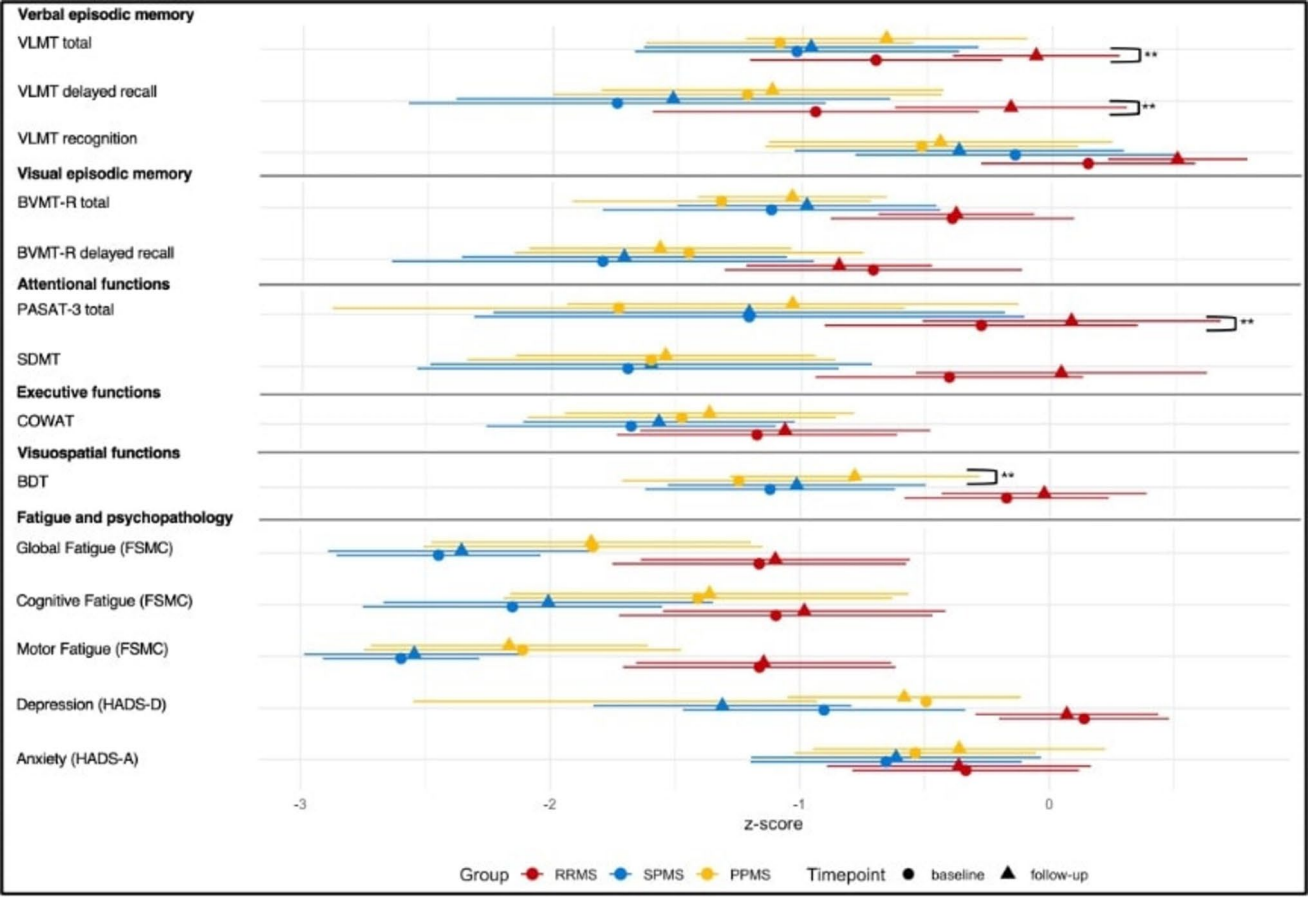
Between-group comparison from baseline to follow-up

For the PPMS subgroup, significant improvements from baseline to follow-up were observed in visuospatial functions (BDT: *t*(20) = − 3.72, *p* = 0.001, d = − 0.812) and in global cognition (*t*(20) = − 2.13, *p* = 0.045, d = − 0.466). In contrast, SPMS patients showed no significant changes from baseline to follow-up (*p* > 0.05) (Fig. 3).

**Fig. 4:**
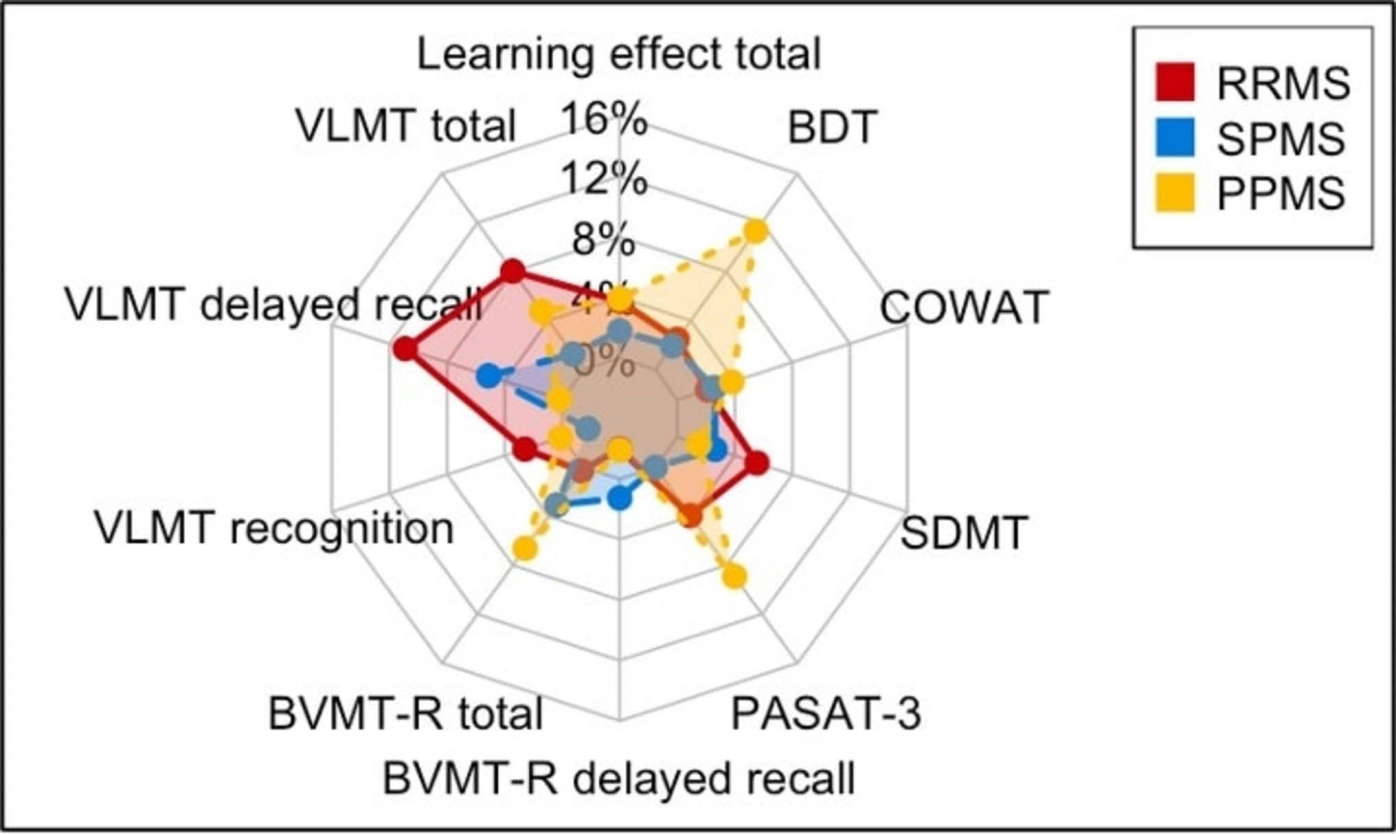
Between-group comparison of learning effects in cognitive domain subtests

RRMS patients achieved the greatest improvement from baseline to follow-up, particularly in VLMT total and VLMT recall scores (statistically significant). PPMS patients exhibited the most pronounced learning effect in the visuospatial BDT test (significant change in within-group comparison; Fig. 4).

## Discussion

Our study examined the cognitive and psycho-behavioral trajectories of clinically defined MS subtypes over one year. The findings highlight significant differences in cognitive functioning, psychopathology, and fatigue among patients with different MS subtypes compared to HC and among MS subtypes themselves. Moreover, our results indicate that the subtype of MS is associated with () specific kinds of cognitive deficits, suggesting the need for subtype-specific therapeutic interventions.

### Between-group comparison of cognition, psychopathology and fatigue at baseline

Cognitive impairment was evident in all MS subtypes at baseline, with patients in the SPMS and PPMS subgroups exhibiting poorer cognitive performance compared with HC. Even in early relapsing–remitting MS (RRMS), impairment was observed in executive functions. Our findings align with the existing literature indicating that cognitive decline is an early and prevalent symptom of MS that is often more severe and more extensive in progressive forms of MS [2, 28]. Deficits in verbal episodic memory, processing speed, attention, and executive function were particularly pronounced in progressive MS. These impairments were evident both at the group level, when SPMS and PPMS were combined into a progressive MS group, and at the subtype level, when distinct patterns of cognitive dysfunction emerged for SPMS and PPMS. Our data suggest an urgent need for early cognitive assessments and targeted interventions for SPMS and PPMS patients, as they may experience more significant neuronal loss and cognitive decline [29] affecting quality of life. Psychopathological symptoms at baseline, especially depression and anxiety, were also significantly elevated in the SPMS group compared with HC, which is in line with previous studies [30]. The complex interrelationship between cognitive impairment and psychological factors suggests that stabilizing mental health and fatigue may attenuate cognitive decline in MS. Elevated fatigue levels were reported across all MS subtypes at baseline, with SPMS patients experiencing the highest levels, aligning with the existing literature on fatigue prevalence and severity[31]. Moreover, our data reinforces the pervasive nature of fatigue in MS and its detrimental impact on quality of life and brain function.

### Between-group comparison of cognition, psychopathology and fatigue at follow-up

Drop-out rate was minimal (1.14%), providing a remarkably complete data set. Specifically, only one participant withdrew from the study due to the long travel distance to the clinic and a reluctance to undergo another round of cognitive testing. Importantly, this participant did not differ significantly from the overall group in terms of psychiatric conditions or other relevant factors. At one year, HC consistently outperformed all MS subtypes, particularly in verbal and visual episodic memory, attentional, executive, and visuospatial functions. The persistent cognitive differences observed between HC and patients with progressive forms of MS highlight the significant cognitive deficits associated with progressive disease. This aligns with existing literature suggesting more pronounced and extensive cognitive impairments in SPMS and PPMS compared with RRMS (at combined group level and subtype level) and HC [2, 10, 28]. Additionally, elevated levels of depressive symptoms and fatigue reported in the progressive MS groups suggest that combined treatment approaches should address both cognitive rehabilitation and emotional well-being [32]. The high fatigue levels reported among all MS subtypes at follow-up highlight the necessity of fatigue management interventions.

### Relationship between cognition, psychopathology and fatigue among MS subtypes

In our study, cognitive function did not consistently correlate with fatigue or psychopathology scores, other than a baseline association observed in RRMS. Our results contrast with an earlier study that showed an association between fatigue and global cognition [35]. However, two other studies also did not show relationships between depression and specific cognitive functions including memory, language, and visuospatial functions [36, 37]. The absence of consistent correlations between cognition and fatigue or psychopathology suggests that heterogenous mechanisms associated with intrinsic disease processes and neurodegeneration may influence cognition, particularly in progressive forms of MS. In contrast to the lack of correlation with cognition, consistent associations between fatigue and psychopathology were observed across all MS subtypes at follow-up, suggesting that these factors may potentiate each other. These results align with existing literature [33, 34]. It may therefore be hypothesized that the relationship between cognitive and psychopathological factors is more complex than previously assumed.

### Within-group comparison of cognition, psychopathology and fatigue from baseline to follow-up

Within-group comparisons from baseline to follow-up showed that the RRMS group displayed cognitive improvement, particularly in tasks assessing verbal episodic memory and attention. The reason for this improvement is unclear and confirmatory data are required. Possible hypotheses include (1) Cognitive recovery may occur in RRMS patients during periods in which MS is stable, particularly as cognitive fatigue showed a slight, though non-significant, decrease from baseline to follow-up. (2) Improvements in the RRMS group might indicate a higher cognitive reserve, as these patients potentially benefitted from repetition effects. (3) The RRMS group might have particularly benefitted from stabilizing treatment effects. In contrast, SPMS and PPMS patients showed no significant improvement, suggesting that disease progression may have influenced their cognitive reserve, impairing their ability to benefit from repetition effects. Additionally, the stability of psychological symptoms and fatigue levels between baseline and follow-up for all groups may indicate that these factors might be entrenched in the disease process, a finding that aligns with existing literature [38].

While our study offers valuable insights, some limitations should be acknowledged. The sample size of 88 participants prevents us from drawing general conclusions from our results. Variations in age among groups at baseline necessitated statistical adjustment; however, age did not significantly correlate with changes over the one-year study period, indicating that it likely did not influence our findings. The one-year follow-up duration may have constrained our ability to detect more pronounced MS-related changes, particularly given the overall low disease activity as reflected by stable EDSS scores. Possible confounding factors such as comorbidities, medications, and lifestyle could not be statistically addressed in this small study. Although parallel test versions were used to mitigate practice effects, a learning effect may have persisted, particularly within the RRMS group, potentially affecting the accuracy of long-term cognitive assessments. In this context, any residual learning effects may primarily be interpreted as a measure of cognitive reserve, which differed between groups. Future research with longer follow-up periods is necessary to provide deeper insights into the long-term progression of cognitive symptoms.

Overall, our study addresses an important gap in the literature by focusing on underrepresented SPMS and PPMS patient groups. By jointly examining cognitive function, fatigue, and psychopathology across all MS subtypes over a one-year period, we were able to make direct comparisons between MS subtypes and offer new insights into the neuropsychological and psychopathological profiles of each subtype. Progressive MS forms exhibited more pronounced and widespread cognitive impairments than RRMS. Fatigue and psychopathological symptoms were present at moderate to high levels and remained stable at the one-year follow-up in all MS groups.

Given the probable interplay between cognitive and psychopathological factors in MS patients, interventions should prioritize both cognitive rehabilitation and psychological support, particularly for progressive MS. Extended follow-up studies are needed to understand long-term cognitive trajectories and refine subtype-specific management and treatment strategies.

## Data Availability

Anonymized data will be shared upon reasonable request from a qualified investigator.
